# A novel protein truncating mutation of TTC8 causes Bardet-Biedl Syndrome (BBS) in a Pakistani family

**DOI:** 10.1590/1678-4685-GMB-2025-0020

**Published:** 2026-02-09

**Authors:** Sana Fatima, Dong Sun, Jianguo Han, Ming Qiu, Safeer Ahmad, Muhammad Zubair, Muhammad Zeeshan Ali, Safdar Abbas, Maria Shafiq, Muhammad Muzammal, Hadia Gul, Jabbar Khan, Shiwei Du, Muzammil Ahmad Khan

**Affiliations:** 1 Gomal University, Gomal Center of Biochemistry and Biotechnology, Dera Ismail Khan, Pakistan.; 2 Shenzhen University, South China Hospital, Medical School, Department of Neurosurgery, Shenzhen, P.R. China.; 3 University of Helsinki, Faculty of Medicine, Research Program for Clinical and Molecular Metabolism, Helsinki, Finland.; 4 Gomal Medical College, Dera Ismail Khan, Pakistan.; 5 Gomal University, Institute of Biological Sciences, Dera Ismail Khan, Pakistan.

**Keywords:** BBS, TTC8, nonsense mutation, Pakistani family, whole-exome sequencing

## Abstract

Bardet-Biedl syndrome (BBS) is a rare ciliopathic disorder that segregates in an autosomal recessive manner. Genetic studies have so far identified 26 BBS-associated genes worldwide. This study analyzed a multiplex consanguineous Pakistani family with Bardet-Biedl syndrome. Genetic analysis was performed using whole-exome sequencing and Sanger sequencing. Additionally, *in silico* predictions were performed for functional characterization of the identified mutation. Whole exome analysis of this family identified a novel nonsense mutation [(NM_144596: exon11:c.C1047G: p.(Tyr349*)] in the 11^th^ exon of *TTC8* gene. The identified mutation presumably leads to removal of four TPR domains and C-terminus portion. Structural analyses of mutant TTC8 protein showed substantial morphologic and interactional variations, suggesting a defective role of the TTC8 protein in BBSome complex and thus its involvement in disease progression. Identification of novel mutation has expanded the mutational spectrum of *TTC8*. Moreover, these findings will help in genotype-phenotype association, prenatal diagnosis and genetic counseling of families at risk of BBS syndrome.

## Introduction

Bardet-Biedl syndrome (BBS) is a rare genetic disorder that segregates in an autosomal-recessive pattern. Clinically, it is categorized as a multisystem abnormality that shows considerable phenotypic heterogeneity. The primary clinical manifestations of BBS include polydactyly, retinitis pigmentosa (RP), renal abnormalities, cognitive weakening, obesity, and hypogonadism (Rao et al., 2023). Developmental delay, endocrine abnormalities, olfactory dysfunction, dental defects, and inherited heart disease are secondary or minor features of BBS (Janssen et al., 2011). Its global prevalence is estimated to be 1 in 150,000 individuals (Azizi et al., 2024). To date, 26 causative genetic factors have been shown to be associated with BBS, emphasizing its genetic diversity (M’hamdi et al., 2014; Liu et al., 2019; Jeziorny et al., 2020; Azizi *et al.,* 2024).

BBS is one of several ciliopathies that is triggered by a malfunction of the primary cilia. Primary cilia are microtubule-based organelles. The apical extensions of cilia are present on virtually all quiescent cell types in mammals (Janssen et al., 2011; Anvarian et al., 2019; Shamseldin et al., 2020). The classification of primary ciliary bodies, as independent organelles, is due to the presence of their outer membrane, which displays unique protein sets and signaling factors (Garcia-Vallve et al., 2000). They act as sensory organelles which coordinate among several signaling pathways such as Hedgehog (Hh), TGF-β, and WNT signaling pathway. These pathways regulate tissue differentiation, cell polarity, migration as well as proliferation during the development of the embryo along with maintaining tissues homeostasis (Harville et al., 2010; Rosengren et al., 2018). The assembly, maintenance, and sensory role of primary cilia depends on BBSome complex, which is composed of BBS1, BBS2, BBS4, BBS5, BBS7, BBS8 (TTC8), BBS9 (PTHB1), and BBS18 (BBIP1) (Loktev et al., 2008; Nachury and Mick, 2019; Domingo-Gallego et al., 2022). BBSome is recruited to the cilium base, where it performs a crucial part in trafficking ciliary proteins through intra-flagellar transport (IFT) machinery. Additionally, it aids in the removal of activated G protein-coupled receptors (GPCRs) and other peripheral membrane proteins from the cilium (Chen et al., 2011; Wingfield et al., 2018). Notably, removal of GPCRs like PTCH1 and SMO, suggests potential regulatory roles in the Hedgehog (Hh) signaling pathway (Ansley et al., 2003; Nachury and Mick, 2019).

The current genetic study describes a consanguineous Pakistani family with BBS syndrome. Molecular investigation in this family revealed a novel nonsense mutation [(NM_144596: exon11:c.C1047G: (p.Tyr349*)] in gene *TTC8*. The identified nonsense mutation predictably causes loss of C-terminus of TTC8 protein, especially TPR domains.

## Subjects and Methods

### Sample collection and clinical phenotyping

The present consanguineous BBS family was recruited from a rural region of KP province in Pakistan. The apparent features of the patients showed polydactyly in hands and feet, obesity, and vision loss, which are common in BBS patients. The family was interviewed about their clinical symptoms, and a family pedigree was drawn. Clinical data was acquired from medical reports of the affected family members. 

### Molecular analysis

Whole exome sequencing (WES) was performed on a single patient (IV-3) using the xGen Exome Research Panel v2.0 Kit (Integrated DNA Technologies, Coralville, IA, USA) to develop a sequencing library. Whereas, paired-end sequencing was performed on NovaSeq 6000 (Illumina, San Diego, CA, USA). The average read depth of target region was 100X. The target enrichment region included exonic sequences and exon-intron boundaries. The BCF (base call files) were converted into FASTQ files using bcl2fastq v2.20.0.422 (https://support.illumina.com/sequencing/sequencing_software/bcl2fastq-conversion-software.html). Afterward, the obtained sequence reads in FASTQ files were aligned with the human reference genome (GRCh37/hg19) via BWA-mem 0.7.17 (arXiv:1303.3997v2 [q-bio. GN]), which generated BAM files. Variant calling was performed on the obtained BAM files to generate VCF (variant call format) files using GATK v.3. Upon variant calling, we obtained more than 30,000 variants. Afterwards, the annotated exome data were then checked to identify the pathogenic variants causing the disease. The variant prioritization was performed by checking the frequency of the variant via online databases including ClinVar, gnomAD, SAGE, GenomeAsia100k, IndiGenomes, and InterVar. The pathogenicity of the candidate variants were investigated using *in silico* tools such as Polyphen2 (http://genetics.bwh.harvard.edu/pph2/), and MutationTaster (https://www.mutationtaster.org/). Once the most probable pathogenic variant was prioritized, its segregation in the whole family was carried out. For PCR amplification of the target region, primers were designed using the Primer3 Plus software (https://www.primer3plus.com/). PCR was used to amplify the target DNA and subsequently Sanger sequencing was performed.

### Protein modeling and docking

To check the drastic consequences of mutation on the configuration and folding of the resultant protein, 3D modeling was performed using the online I-Tasser tool (https://zhanggroup.org/I-TASSER/). The obtained model was visualized using the UCSF Chimera software (https://www.cgl.ucsf.edu/chimera/). To check for interactional changes in the mutant protein, the close interactor of TTC8 was determined using the STRING database (https://string-db.org/). Docking was then performed for both normal and mutant-interactor by using Cluspro (https://cluspro.org), and then models were visualized via LigPlot (https://www.ebi.ac.uk/thornton-srv/software/LigPlus/).

Ethical approval for this study was granted by the “Institutional Review Board of Gomal University, Dera Ismail Khan (04/ERB/GU dated 16/10/2017).” Blood samples were collected from available normal and affected individuals, after obtaining the written consent from family’s elders.

## Results

### Clinical findings

**Figure 1 -:**
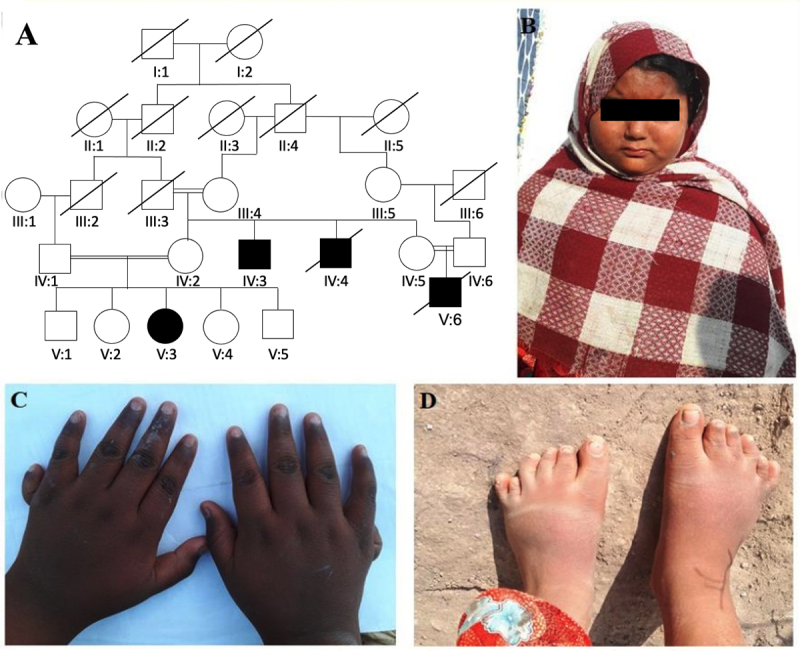
(A) Family pedigree shows autosomal recessive inheritance. (B) Facial appearance of the affected member (IV-3). (C-D) Hands and feet of the affected member (IV-3) displaying bilateral postaxial polydactyly.

The recruited family comprise of four affected members (IV-3, IV-4, V-3 and V-6) from three different loops. The pedigree analysis indicated an autosomal recessive mode of disease segregation (Figure 1 A). All affected individuals were suffering from cognitive impairment, obesity, vision impairment, and gastrointestinal problems, while postaxial polydactyly (PAPA) was observed in only two patients (IV-3 and IV-6). Patient IV-3 displayed bilateral PAPA of both hands and feet (Figure 1 B-D ), whereas the affected individual IV-6 demonstrated bilateral PAPA of only hands (not shown in the figure). The clinical details of the affected individuals are summarized in Table 1.

**Table 1 -:** Clinical findings of affected members.

Clinical findings	Pedigree: IV-3	Pedigree: V-3	Pedigree: V-6
Age (years) (at the time of visit)	35	10	5
Sex	Male	Female	Male
Weight (kg)	85	65	Unknown
Obesity	Yes	Yes	Yes
Polydactyly	No	Yes/ PAPA in both hands as well as feet	Yes/PAPA in both hands
Night blindness	No	Yes	Yes
Mental retardation	Yes	Yes	Yes
Hypogonadism	Unknown	Unknown	Unknown
Renal abnormality	No	No	No
Speech disability	No	No	No
Dental defects	No	No	No
Gastrointestinal problems	Yes	Yes	Yes
Heart defects	No	No	No

PAPA: Postaxial polydactyly type A.

### Molecular findings

**Figure 2 -:**
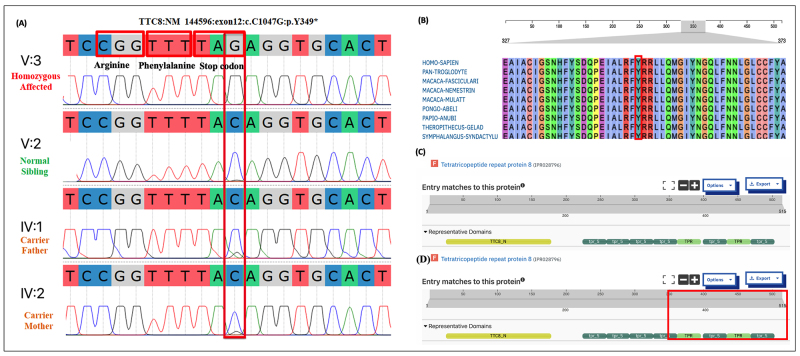
(A) Sequence chromatogram of TTC8 12^th^ exon, representing member V:3 as affected by homozygous mutant alleles, V-2 as homozygous unaffected, and IV-1 and IV-2 as carriers with heterozygous mutant alleles. The vertical red rectangular box shows the point of the substituted bases, whereas the small red rectangular boxes show the 3-code translation of the proteins. (B) Clustal Omega showing a highly conserved region around Tyr349 in TTC8 among different species. (C) TTC8 (normal) protein domain predicted via InterPro (entry ID: IPR028796) (D) Point of truncation is shown by the rectangular box, which represents the truncation of protein and loss of four TRP domains.

Analysis of whole-exome data from patient IV-3 identified a novel nonsense mutation [(NM_144596:c.C1047G: p.(Tyr349*)] in the 11th exon of *TTC8* gene. The creation of premature stop codon suggestively produces 349 amino acid truncated protein with loss of C-terminus region, including four TPR domains. This mutation was not found in, HGMD, ClinVar, gnomAD, South Asian Genome and Exome (SAGE), IndiGenomes, or GenomeAsia100k. InterVar classified this variant as pathogenic, according to ACMG classification. Mutation Taster predicted the identified variant as disease causing, whereas the Combined Annotation Dependent Depletion (CADD) Phred score was 40 (GRCh37-v1.6). The NMDetective-A score for this variant was determined 0.59, indicating a strong likelihood of mRNA degradation via nonsense-mediated decay (NMD). Segregation analysis of this mutation was validated and revealed that the patient was homozygous with respect to G allele, while normal persons were either heterozygous such as C/G or homozygous with respect to C allele. The sequence chromatogram analysis showed that V-3 (affected member) is homozygous whereas IV-1 and IV-2 (carrier members) are heterozygous (Figure 2 A ).

### *In silico* findings

*Conservation of amino-acid*

Multiple sequence alignment was performed using Clustal Omega (https://www.ebi.ac.uk/jdispatcher/msa/clustalo), which showed that this region, surrounding the Tyr349 (TTC8), is highly conserved across various species. The protein domains database InterPro (https://www.ebi.ac.uk/interpro/) showed that TTC8 protein harbor nine functional domains including TTC8_N domain and eight TPR domains. The structural analysis of mutant TTC8 protein indicates that p.(Tyr349*) predictably lead to truncation of the protein, which causes loss of four TPR domains. TPR domains play significant role in establishing interaction of TTC8 protein with other proteins (Figure 2 B_D).

*Protein Modelling*

**Figure 3 -:**
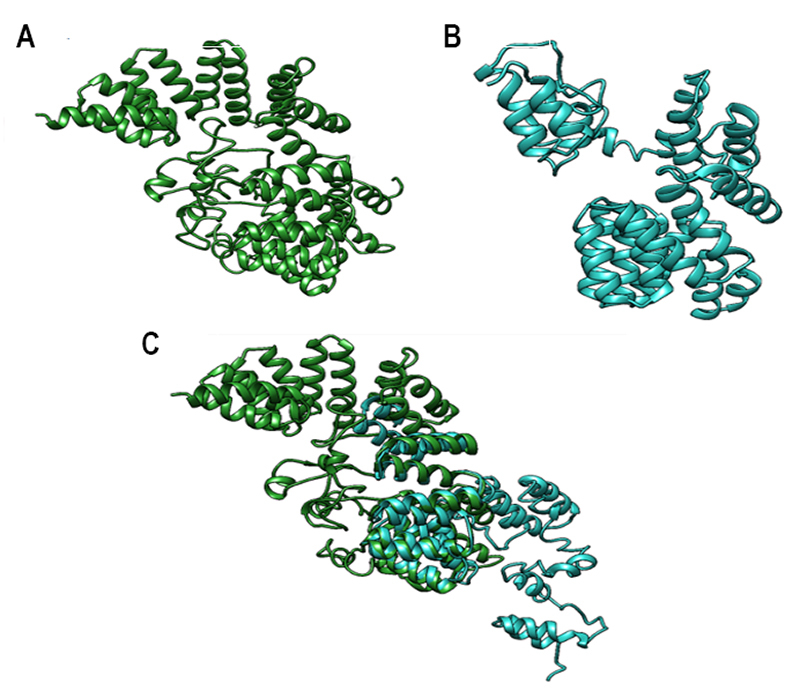
Three-Dimentional assembly of the TTC8 protein was projected using I-TASSER (A) Normal TTC8 (B) Mutant TTC8 (C) Superimposed structure of normal and mutant TTC8.

Three-dimensional (3-D) structures of the normal and mutant TTC8 protein were superimposed for comparison of folding pattern. The analysis determined a percent identity of 34.67, which indicated gross structural dissimilarity (Figure 3 A-C ).

*Protein-Protein Interaction*

**Figure 4 -:**
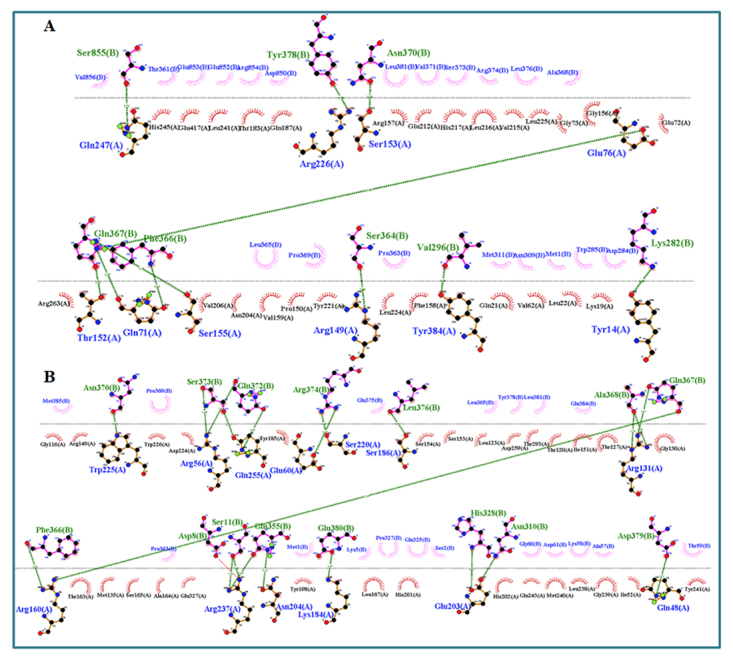
Interaction among normal as well as mutant TTC8 with BBS9 as a close interactor. (A) Ten residues (chain A) of the normal TTC8 interact with eight residues (chain B) of BBS9 by making 11 hydrogen bonds. (B) Mutant TTC8 make interaction via its 13 residues (chain A) with 15 residues (chain B) of BBS9 by making 21 hydrogen bonds and 1 salt bridge.

The comparative docking results showed a substantial alteration in the interaction pattern of normal and mutant TTC8 protein with its close interactor. BBS9 was predicted to be the close interactor of TTC8 with a probability score of 0.999. Docking of normal TTC8 revealed that 10 residues of TTC8 interact with eight residues of BBS9 by making 11 hydrogen bonds, while 13 residues of mutant TTC8 interacted with 15 residues of BBS9 through 21 hydrogen bonds and one salt bridge (Figure 4 A-B ).

## Discussion

The present study involved a consanguineous Pakistani family demonstrating a ciliopathic condition, namely BBS syndrome. Genetic studies have found 26 genes to be associated with BBS (data accessed from OMIM on 23/05/24). The encoded proteins of these genes are associated with normal morphology and physiology of primary cilia (Harville et al., 2010). Primary cilia has a crucial role in the functioning of retinal photoreceptor cells and the transportation of various molecules in these cells. Ciliary dysfunction may lead to the degeneration of retinal cells, obesity, cerebral anomalies, renal diseases, diabetes and limb defects (Berbari et al., 2008). BBS genes mainly participate in ciliary functions and intra-flagellar transportation (IFT). Seven of these genes including BBS1, BBS2, BBS4, BBS5, BBS7, BBS8 (*TTC8*), and BBS9 contribute to the development of the BBSome complex, while BBS6, BBS10, and BBS12 make proteins for chaperone complex of BBS, which is necessary for the regulation and maintenance of the BBSome (Klein and Ammann, 1969).

The current study involved a multiplex BBS family presenting night blindness, obesity, intellectual disability, and postaxial polydactyly. WES determined a novel nonsense mutation [(NM_144596:c.C1047G: p.(Tyr349*)] in the 12^th^ exon of tetratricopeptide repeat domain 8 (*TTC8*) gene. Due to premature stop codon, protein truncation occurs, which presumably remove four TPR domains towards the C-terminus. The tetratricopeptide repeat domain 8 gene is present on chromosome 14q32.1, comprised of 15 exons, and encodes a 531 amino acids long protein. TTC8 protein is a constituent of the BBSome complex. The TPR (tetratricopeptide repeat) domain (Sikorski et al., 1990) is a degenerate, 34-amino acid, repeated motif that is commonly found in multiple species. Within the cell, proteins harboring TPR domains are generally situated in a variety of subcellular compartments, including cytoplasm, nucleus, and mitochondria (Goebl and Yanagida, 1991). The TPR proteins are generally involved in cell-cycle control, neurogenesis, transcription repression, mitochondrial and peroxisomal protein transport, stress response and protein kinase inhibition. TPR repeats facilitate the assembly of multiprotein complexes via protein-protein interactions (Lamb et al., 1995). Hence, it is speculated that TTC8 may be involved in BBSome formation. It shares similarities with BBS4 protein which contains eight TPR domains that participate in interactions among proteins. The *TTC8* is expressed only in neurons, having cilia and contains RFX-binding elements. RFX acts as a transcription factor that controls the gene expression linked to the genesis of cilia and intra-flagellar transportation (IFT). Additionally, *TTC8* has similarities to a prokaryotic domain called pilF, which participates in twitching mobility along with the assembly of type-IV pilus (Ansley et al., 2003; Stoetzel et al., 2006; Ullah et al., 2017). 

To date, at least 34 mutations in the *TTC8* gene have been identified (data accessed from HGMD on 23/05/24). Out of these mutations, 23 are associated with BBS, and six are linked to non-syndromic retinitis pigmentosa (RP) (Riazuddin et al., 2010; Wang et al., 2014; van Huet et al., 2015; Jespersgaard et al., 2019; Goyal and Vanita, 2022) and four with rod-cone dystrophy, dysmorphic syndrome, breast cancer, and sex development disorder respectively (Shahi et al., 2019; Yohe et al., 2020; Abualsaud et al., 2021; Saini et al., 2022). Genotype-phenotype association has revealed that TTC8-associated BBS disorder mostly involves intellectual disability, polydactyly, retinal degeneration and obesity. However, renal anomalies and hypogonadism have also been seen occasionally. The clinical description of *TTC8* gene mutations associated with BBS among various ethnicities is summarized in Table 2. Previous studies have demonstrated only two *TTC8* variants in Pakistani families suffering from BBS (Ansley et al., 2003; Redin et al., 2012; Ullah et al., 2017; Nasser et al., 2022) and one variant with RP (Riazuddin *et al.,* 2010). Rao *et al.,* studied 12 BBS families comprising of 31 affected individuals from various regions of Pakistan. These individuals carried different homozygous mutations in BBS genes. Their investigation revealed that the frequent BBS-associated mutants in Pakistani national patients involve BBS6/*MKKS* alleles, which were found in 41.6 % (5/12) of the families (Sato et al., 2019; Rao *et al.,* 2023). Our study presented a third Pakistani family harboring a novel *TTC8* gene variant causing BBS. Additionally, our patients were also presenting gastrointestinal complications, which adds a new phenotypic variant in the clinical spectrum of *TTC8*-associated BBS syndrome. In a broader sense, these findings contribute to expanding the genetic and clinical spectrum of the *TTC8* gene associated with BBS. The study will help to explore the underlying genetic mechanisms of ciliopathies, provide opportunities for carrier screening and genetic counseling families suffering from BBS.

**Table 2 -:** Reported variants of *TTC8* gene associated with BBS cases.

Variant	Family Origin	Patient/Family ID (s)	Phenotypes	Secondary signs	Reference
c.1047G>C; p.Tyr349*	Pakistan	III-3	OB, MR	Gastrointestinal complications	Present study
		IV-3	PD, OD, OB, MR		
		IV-6	PD, OD, OB, MR		
c.694G>A; p.Gly232Arg	German	BBS67	PD, OD, OB, MR	Developmental delay, Brachydactyly, Vitamin D deficiency,	Nasser *et al.,* 2022
c.69del; p.Cys23Trpfs*31 & c.(768+1_769-1)_ (879+1_880-1)del	Spanish	P144	PD, RA	No	Domingo-Gallego et al., 2022
c.489G>A; p.Thr163=	Polish	BBS No.3	PD, OD, OB, RA, MR	Cardiovascular Problems, Hearing Loss, dyslipidemia	Jeziorny *et al.,* 2020
c.725G>A; p.Arg242His		BBS No.10	OB, RA, MR	Diabetes mellitus, Hearing Loss hepatic steatosis, dyslipidemia	
c.226C>T;p.Q76* & c.309_310insTA, p.T103fs	Japanese	NA	PD, OD, OB, RA	Hirschsprung disease, peculiar tolerance for glucose, exotropia, hypertension	Sato *et al.,* 2019
c.769-2A>G (Splice site)	East Asian	10-1	PD, OD, OB	Vitiligo, cryptorchidism	Liu *et al.,* 2019
c.1347G>C; p.Gln449His	Pakistan	IV-1	PD, OD, OB, HP, MR(CI)	Clinodactyly	Ullah *et al.,* 2017
		IV-2	PD, OD, OB, HP, MR(CI)	Clinodactyly	
		IV-6	PD, OD, OB, MR(CI)	NA	
c.459+1G>A (Splice site)	Tunisian	BBS-F14A	PD, OD, OB, RA, HP	Dental abnormalities, hypertension	M’hamdi *et al.,* 2014
c.329+1G>A (Splice site)	Tunisian	P14	NA	NA	Redin *et al.,* 2012
IVS2+1G>A (Splice site)	NA	A2506	PD, OD, OB(?), MR	Nasal cephalocele, Asthma	Janssen *et al.,* 2011
c.485del; p.Gly162fs*4 & c.1000del; p.Ile334fs*1	Hispanic	A2513	PD, OD, OB, RA, HP, MR	Gall stones, Fatty liver	-
c.1011C>G; p.Phe337Leu	Tunisian	057015	NA	NA	Chen *et al.,* 2011
c.405G>A; p.Met135IIe	Arabic	E059	NA	NA	
c.122G>A; p.Trp41*	Turkey	A2011-II1	PD, OD, OB, HP	Yes (But no details are known)	Harville *et al.,* 2010
c.355_356ins GGTGGAAGGCCAGGCA; p.Thr124Argfs*43	Tunisian	57006	NA	NA	Smaoui *et al.,* 2006
		57019	NA	NA	
		57009	NA	NA	
c.459G>A, p.Thr153=	North African	II.3	PD, OD, MR(CI)	Micropenis	Stoetzel *et al.,* 2006
		II.5	PD, OD, RA	Hydrometrocolpos	
		II.6	PD, OD, RA	NA	
IVS6+1G>A (Splice site)	Lebanese	NA	NA	NA	
IVS6+1-2delGT (Splice site)	Caucasian	NA	NA	NA	
187-188delEY; 6bp Inframe deletion 187-188delEY; 6bp Inframe deletion	Saudi Arabian	Fmaily KK049 (1)	PD, OD, OB, HP, MR(SI)	Brachycephaly, Developmental delay	Ansley *et al.,* 2003
		Fmaily KK049 (2)	PD, OD, OB, HP, MR(SI)	Brachycephaly, Developmental delay	
		Fmaily KK049 (3)	PD, OD, OB, MR(SI)	Developmental delay, deafness brachycephaly	
		Family KK069 (1)	PD, OD, OB, MR(SI)	Developmental delay, hypospadias, brachycephaly	
		Family KK069 (2)	PD, OD, OB, MR(SI)	Developmental delay, asthma, brachycephaly	
IVS10+2_4delTGC (Splice site)	Pakistan	Family BB12 (1)	PD, OD, OB, HP, MR(SI)	Brachycephaly, Developmental delay	
		Family BB12 (2)	PD, OD, OB, HP, MR(SI)	Developmental delay, Situs inversus brachycephaly	
		Family BB12 (3)	PD, OD, OB, HP, MR(SI)	Developmental delay, hemophilia, brachycephaly	

*PD: Polydactyly, OD: Ophthalmic Defect, OB: Obesity, RA: Renal Anomalies, HP: Hypogonadism, MR: Mental Retardation, CI: Cognitive Impairment, SI: Speech Impediment, NA: Not Applicable, *: Terminated.

## Conclusions

Herein this report, we described a consanguineous Pakistani family with BBS syndrome. Molecular investigation in this family revealed a novel nonsense mutation [(NM_144596: exon11:c.C1047G: (p.Tyr349*)] in gene *TTC8*. The identified nonsense mutation predictably causes loss of C-terminus of TTC8 protein, especially TPR domains. Detailed phenotyping of previously reported *TTC8* mutations and their associated clinical features confirm inter-/intra-familial clinico-genetic heterogeneity. The current genetic study and genotype-phenotype association would be helpful in molecular diagnosis and genetic counseling of families at risk of ciliopathic disorders.

## Data Availability

 Additional Data is stored and will be provided on request.
